# Investigating the spatial limits of somatotopic and depth-dependent sensory discrimination stimuli in rats via intracortical microstimulation

**DOI:** 10.3389/fnins.2025.1602996

**Published:** 2025-05-14

**Authors:** Thomas J. Smith, Hari Srinivasan, Madison Jiang, Ghazaal Tahmasebi, Sophia Vargas, Luisa R. Villafranca, Shreya Tirumala Kumara, Ashlynn Ogundipe, Ajaree Massaquoi, Shreya Chandna, Yovia Mehretab, Riya Shipurkar, Pegah Haghighi, Stuart F. Cogan, Ana G. Hernandez-Reynoso, Joseph J. Pancrazio

**Affiliations:** ^1^School of Behavioral and Brain Sciences, The University of Texas at Dallas, Richardson, TX, United States; ^2^Department of Bioengineering, The University of Texas at Dallas, Richardson, TX, United States; ^3^Department of Biology, The University of Texas at Dallas, Richardson, TX, United States; ^4^Department of Healthcare Studies, The University of Texas at Dallas, Richardson, TX, United States; ^5^Department of Biomedical Engineering, Case Western Reserve University, Cleveland, OH, United States

**Keywords:** intracortical microstimulation, sensory discrimination, rodent, somatosensory cortex, behavior, microelectrode arrays

## Abstract

The somatosensory cortex can be electrically stimulated via intracortical microelectrode arrays (MEAs) to induce a range of vibrotactile sensations. While previous studies have employed multi-shank MEA configurations to map somatotopic relationships, the influence of cortical depth on sensory discrimination remains relatively unexplored. In this study, we introduce a novel approach for investigating the spatial limits of stimulation-evoked sensory discrimination based on cortical depth and somatotopic relationships in rodents. To achieve this, we implanted single-shank and four-shank 16-channel MEAs into the primary somatosensory cortex of male rats. Then, we defined distinct stimulation patterns for comparison, each consisting of four simultaneously stimulated electrode sites separated along the length of the single-shank device or between shanks for the four-shank device. Next, we utilized a nose-poking, two-choice sensory discrimination task to evaluate each rat’s ability to accurately differentiate between these patterns. We demonstrate that the rats were able to reliably discriminate between the most superficial (450–750 μm) and deepest (1650–1950 μm) single-shank patterns with 90% accuracy, whereas discrimination between the most superficial and next adjacent pattern (650–950 μm) significantly dropped to 53% (*p* < 0.05). Similarly, in the four-shank group, discrimination accuracy was 88% for the furthest pattern pairs (375 μm difference) but significantly fell to 62% (*p* < 0.05) for the closest pairs (125 μm difference). Overall, the single-shank subjects could robustly differentiate between stimuli separated by 800 μm along a cortical column whereas, the multi-shank animals could robustly differentiate between stimuli delivered from shanks separated by 250 μm. Results showed that when spatial distances between stimuli patterns were decreased, the rats had reduced discriminable accuracy, suggesting greater difficulty when differentiating closely positioned stimuli. To better understand the single-shank results, we also utilized computational modeling to compare our in-vivo results against neuronal activation volumes presented in a biophysically realistic model of the somatosensory cortex. These simulations displayed overlapping volumes of activated neurons via antidromic propagation of axons for the closest pattern pair, potentially influencing discriminable limits. This work, which offers insight into how the physical separation of stimulating microelectrode sites maps to discernable percepts, informs the design considerations for future intracortical microstimulation arrays.

## 1 Introduction

Intracortical microstimulation (ICMS) of the primary somatosensory cortex has been explored as a method for restoring vibrotactile sensations in neuroprosthetic applications (Callier et al., 2015; Flesher et al., 2016; Hughes et al., 2021a,b; Lycke et al., 2023). When delivering electrical stimulation via microelectrode arrays (MEAs), ICMS can evoke a wide range of sensory percepts, providing an alternative means of tactile feedback for individuals with sensory deficits (Callier et al., 2015; Kim et al., 2015a; Armenta Salas et al., 2018; Hughes et al., 2021b). One of the most commonly utilized MEA devices for clinical study is the traditional Utah-style array, comprised of fixed-length shanks arranged in a grid-like fashion with a 400 μm pitch between each shank (Flesher et al., 2016; Armenta Salas et al., 2018; Hughes et al., 2021a). During ICMS studies, researchers have used these arrays to map the perceptual boundaries of somatotopic representation in the brain across various regions of the body (Greenspon et al., 2024; Verbaarschot et al., 2024; Valle et al., 2025). However, due to their fixed-length and equidistant shank design, the relationship of cortical depth on sensory discrimination has remained largely unexplored. Although Utah arrays can be fabricated with a slanted design, the electrodes are arranged in different cortical columns. It is unknown whether the discriminable limits of ICMS-evoked percepts are more constrained by cortical depth or lateral spacing between adjacent columns. To address these knowledge gaps, we built upon our validated preclinical behavioral paradigm (Smith et al., 2023) to investigate how spatial separation between ICMS patterns affects discrimination accuracy and reaction time performance. In this study, we hypothesized that spatially closer ICMS pattern pairs would be more difficult for an animal to distinguish between than patterns that were further apart. Here, male Sprague–Dawley rats (*N* = 8) were implanted with either a single-shank (*n* = 4) or four-shank (*n* = 4) 16-channel MEA into the primary somatosensory cortex and trained to participate in a novel nose-poking two-choice sensory discrimination paradigm. In total, the single-shank group was evaluated on five different stimulation patterns spanning various cortical depths along the shank, while the four-shank group was assessed on four different stimulation patterns separated laterally by a 125 μm inter-shank pitch.

To further interpret the in-vivo discrimination results, we modified an established biophysically realistic computational model of the somatosensory cortex (Kumaravelu et al., 2022) to simulate our multi-channel ICMS. This model simulates ICMS-evoked neural activation across cortical layers 1–6, incorporating detailed neuronal morphologies and axonal projections to predict how different stimulation parameters may influence spatial activation patterns. By applying this model, we asked whether perceptual differences observed in the behavioral paradigm task would be due to distinct neural activation volumes or overlapping spatial recruitment. We simulated various current point source ICMS stimuli at depths corresponding to electrodes sites in our single-shank patterns and then quantified the resulting volume overlap by identifying shared axonal activation sites that initiated antidromic propagation toward the soma between ICMS pattern pairs.

Overall, our results demonstrate that rats were able to successfully discriminate spatially distinct ICMS patterns with accuracies as high as 90% and inter-shank distances nearly half that of the Utah array. As expected, accuracy for both groups declined as spatial proximity between stimulation sites decreased, suggesting that overlapping neural activation may limit perceptual differentiation. Computational modeling reinforced this interpretation, revealing that adjacent depth-dependent ICMS patterns exhibited substantial overlaps in neuronal activation, whereas spatially distinct patterns maintained relatively distinguishable separation volumes. These findings emphasize the importance of spatial selectivity in ICMS applications and suggest that depth-dependent discrimination may impose greater constraints on perceptual resolution than lateral separation across cortical columns. By establishing a behavioral framework for assessing discrimination limits, our findings offer valuable insights for optimizing MEA design in neurostimulation applications aimed at restoring selective sensory percepts and furthering our understanding of stimulation-evoked cortical processing.

## 2 Materials and methods

### 2.1 Animal use

All animals were utilized in accordance with procedures and guidelines approved by The University of Texas at Dallas IACUC (protocol #21–15). In this study, we used eight (*N* = 8) male Sprague–Dawley rats (Charles River Laboratories Inc., Houston, TX, United States) that were single-housed in standard home cages under a reverse 12-h light/dark cycle with lights off at 6 a.m. To promote behavioral engagement, we implemented mild food deprivation and welfare protocols detailed in (Smith et al., 2023), maintaining each animal at 90% + of its free-feeding weight four days per week, with free feeding allowed on weekends and ad libitum access to water at all times. Additionally, we trained the animals to consume dustless reward pellets (F0021, Bio-Serv, Flemington, NJ, United States) as conditioned rewards during behavioral sessions. When supplemented with standard rodent feed (5LL2-Prolab^®^ RMH 1800, LabDiet, St. Louis, MO, United States), this regimen ensured balanced nutrition despite controlled deprivation. If any of the animals were found to be underweight, then additional feed was provided, and the animal would be excluded from behavioral testing until recovered. Additionally, if an animal proceeded to become insensitive to any control stimuli, then they were excluded from the study.

**Figure 1:**
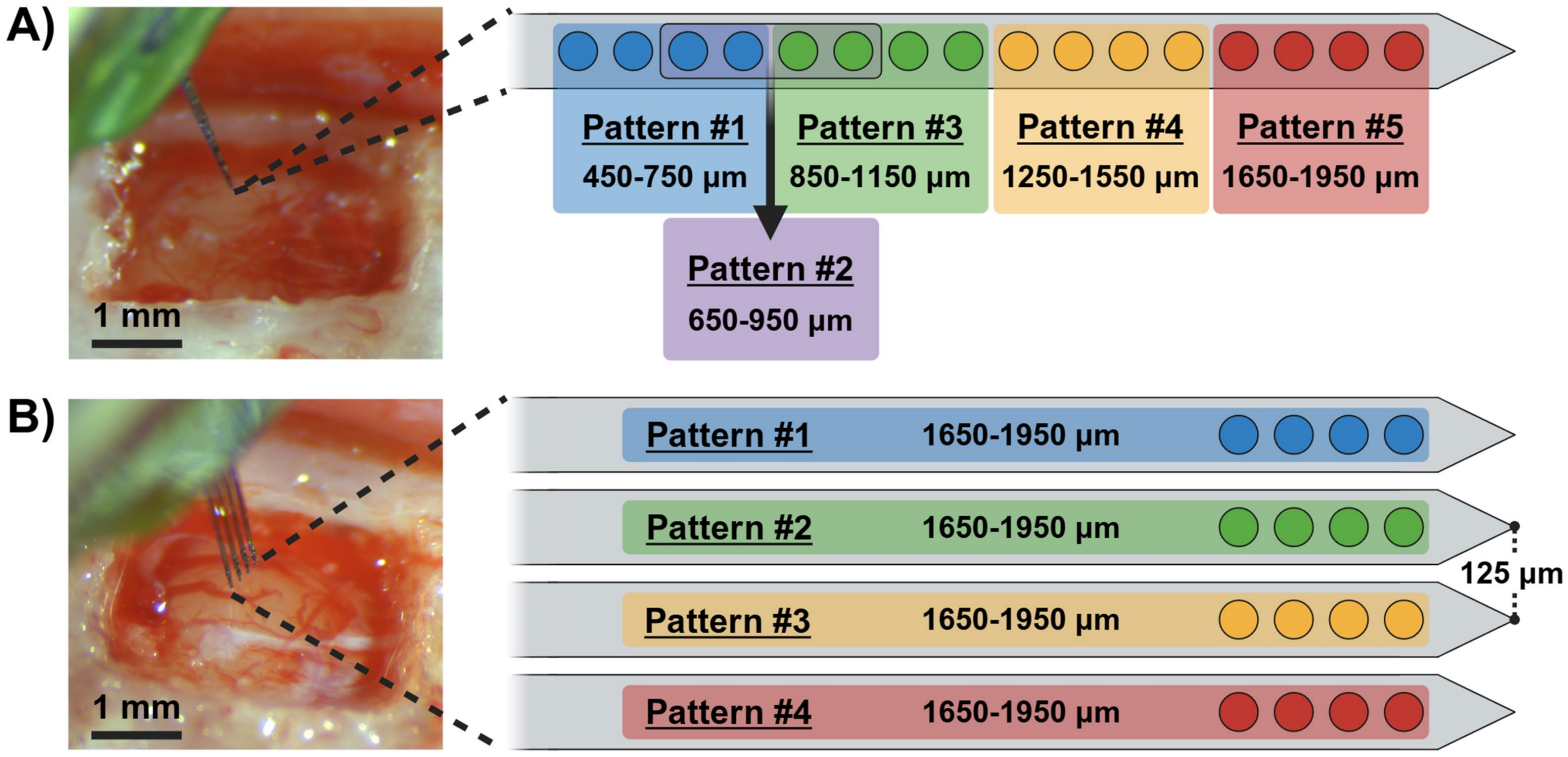
Microelectrode arrays and stimulation pattern groups. Pre-implanted surgical images of single-shank **(A)** and four-shank **(B)** microelectrode arrays with schematics outlining their respective ICMS pattern groups and corresponding post-implantation electrode site depths.

For behavioral investigations, we randomly assigned the animals to one of two groups (single-shank or four-shank) and implanted each with a 15 μm-thick commercially available MEA targeting their primary somatosensory cortex left forelimb region (S1FL; AP: −0.5 mm and ML: 4 mm relative to bregma) at a depth of ~2 mm. Surgical procedures were performed in accordance with (Smith et al., 2023). Briefly, the animals were anesthetized with (1.8–2.5%) isoflurane, placed in a stereotaxic frame, and monitored for vital signs through the procedure. After the initial incision, the skull was leveled within a range < ±0.1 mm between bregma and lambda to promote consistent implantation orientation perpendicular to the brain’s surface. Later, a craniotomy and durotomy was performed over the primary somatosensory cortex, and either a single-shank or four-shank MEA was inserted perpendicular to the cortical surface using a precision-controlled inserter (NeuralGlider, Actuated Medical, Inc., Ann Arbor, MI, United States) with vibrational actuation on to mitigate shank deflection (Wang et al., 2021). After securing the implant with dental cement and closing the wound, antibiotic and analgesic treatments were provided. We implanted the first group (*n* = 4) with a single-shank MEA (Figure 1A; A1x16–3 mm–100–703-CM16LP, NeuroNexus Technologies Inc., Ann Arbor, MI, United States), while the second group (*n* = 4) received a four-shank MEA (Figure 1B; A4x4-3 mm-100-125-703-CM16LP, NeuroNexus Technologies Inc.). Both devices contained sixteen 703 μm^2^ activated iridium electrode sites, equally spaced 100 μm apart along the midline of a 3 mm-long shank, starting 50 μm from their respective tips. In contrast to the single-shank device which included all 16 channels on one shank, the four-shank MEA distributed its channels across four sites per shank with the shanks separated by a 125 μm pitch (Figure 1B). For this work, the single-shank group was employed to investigate the limits of sensory discrimination evoked by ICMS in relation to cortical depth, while the four-shank group explored discrimination limits across adjacent cortical columns.

### 2.2 ICMS parameters and patterns

Stimulation was delivered via an external stimulator (PlexStim, Plexon Inc., Dallas, TX, United States) to each animal using monopolar, charge-balanced, and symmetrical biphasic waveforms with a cathodal-leading phase at a frequency of 320 Hz. Furthermore, each pulse had a duration of 200 μs per phase, separated by a 40 μs interphase delay, and was presented as a 650 ms pulse train (208 pulse-pairs) as demonstrated in previous studies (Urdaneta et al., 2021; Smith et al., 2023). Current amplitudes ranged from 0 to 100 μA (0–20 nC/ph) per electrode, with maximum charge limits set from established literature using similar methods (Urdaneta et al., 2021; Kunigk et al., 2022). Seven to twelve days after MEA implantation, baseline naïve perception thresholds, as described in Smith et al. (2023), were measured to assess ICMS magnitudes suitable for behavioral training as conditioned stimuli as well as confirm implantation at the putative S1FL location via paw withdrawal during initial stimulation trials prior to the behavioral testing (Smith et al., 2023). These paw withdrawals and naïve thresholds were established for each individual animal with various combinations of four simultaneously stimulated channels that were differentially defined across MEA type. In this work, four-channel groupings were used to significantly reduce the per-site charge required for producing reliable percepts (Kunigk et al., 2022) while also maintaining sufficient specificity for non-overlapping channels in both array types. Here, these combinations will be referred to as ICMS “patterns” with channel distributions outlined in Figures 1A,B. In total, the single-shank arrays were stimulated using five different patterns targeting various depths along the shank from superficial to deep cortical ranges. In contrast, the four-shank arrays utilized four different patterns at similar depths, but were constrained to each individual shank.

### 2.3 Behavioral apparatus

**Figure 2:**
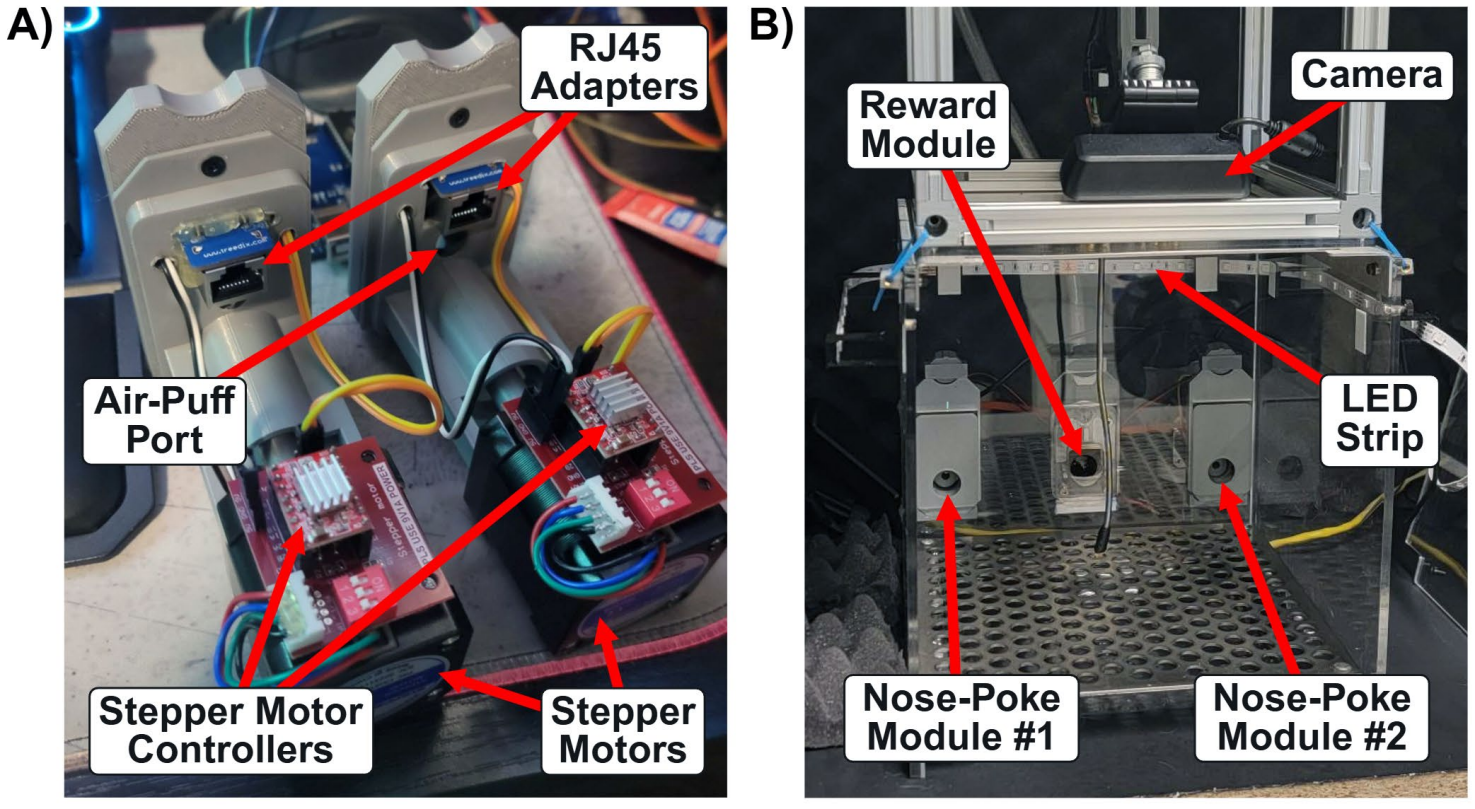
Nose-poke modules and behavioral apparatus. **(A)** Custom nose-poke modules used for sensory discrimination training. Each module includes a stepper motor with an individual controller to drive a retractable plug, an air-puff port for delivering mild punishments, RJ45 adapters for interfacing with an Arduino via ethernet cables, and infrared break-beam sensors for detecting nose-poke interactions. **(B)** Interior view of the behavioral chamber, showing the placement of both nose-poke modules, the centered reward module, and other peripherals.

Behavioral experiments were conducted in a commercially available operant conditioning chamber (OmniTrak, Vulintus Inc.), custom-configured for an ICMS-based nose-poke Go/No-Go paradigm. Comprehensive details on the assembly, hardware, and software implementation of the core components have been previously documented (Smith et al., 2023, 2024a,b). However, to accommodate for a two-choice sensory discrimination task, dual nose-poke modules were implemented opposed to just one module (Figure 2). Furthermore, each nose-poke module was also modified to include an infrared break-beam sensor (Product ID: 2167, Adafruit Industries, NY, United States) for tracking nose-poke interactions, a port for delivering mild air-puff punishments to the nose, and a retractable plugging mechanism controlled by a stepper motor (17HS19-2004S, OSM Technology Co., Ltd.) to regulate hole access during training sequences (Figure 2A). When mounted on either side of the centrally positioned reward module, this configuration enabled animals to associate specific ICMS patterns with individual nose-poke holes (Figure 2B). Here, low torque stepper motors were specifically chosen over a door-like system to allow the animal to be gently pushed out of the hole during pattern association training without the risk of pinching its nose or whiskers mid-session. Finally, signal control for each module was relayed to an Arduino microcontroller and Dell precision 5,860 workstation computer using an RJ45 (ethernet) adapter (B08HGS5G8N, Treedix) and breakout board (43237–2, Electronics-Salon).

### 2.4 Behavioral paradigm

**Figure 3:**
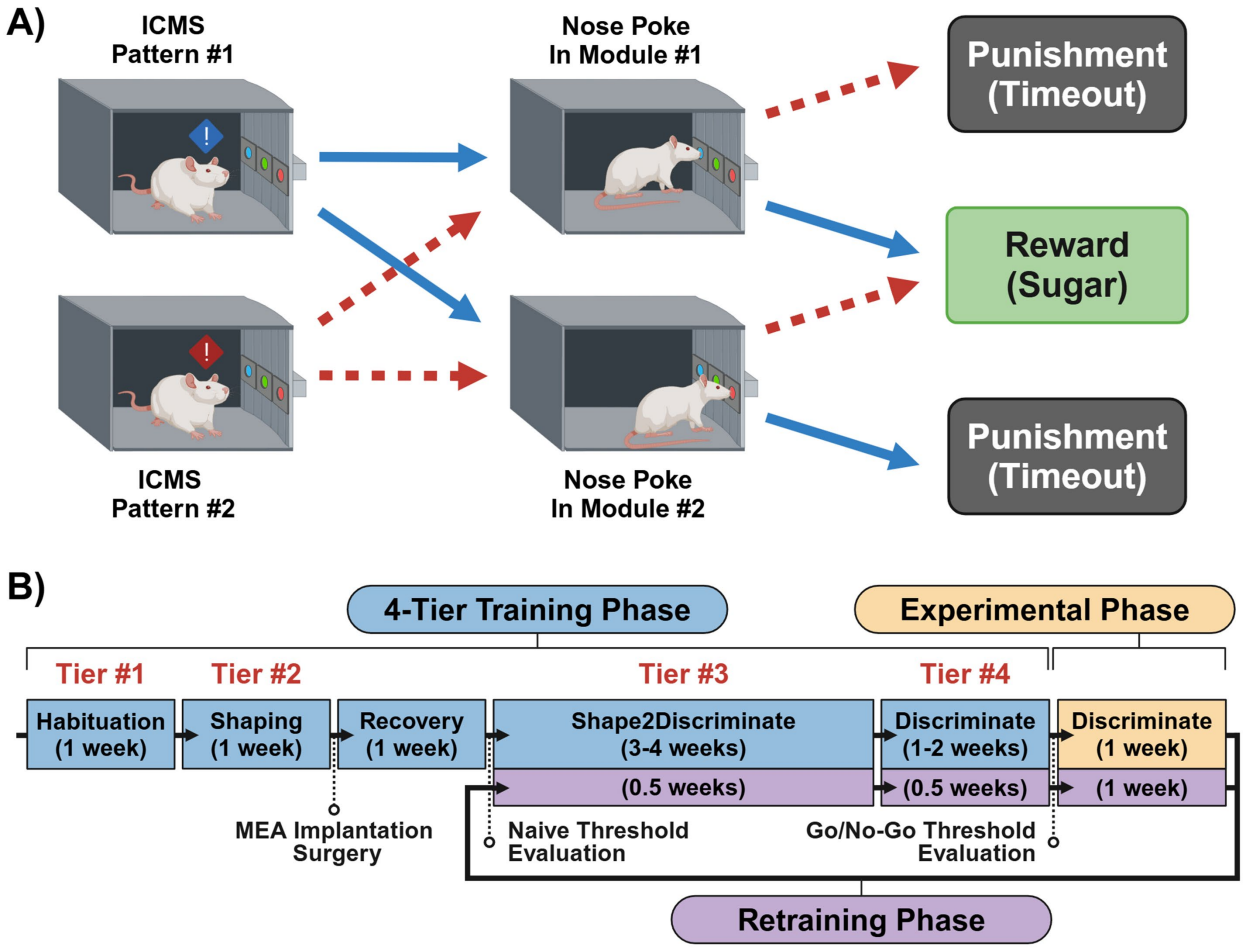
Two-choice sensory discrimination paradigm and training timeline. **(A)** Diagram of the ICMS two-choice sensory discrimination task, illustrating the stepwise processes and expected outcomes that may occur when presenting one of two differing ICMS patterns to an animal. **(B)** Detailed timeline of the experimental tasks and procedures for training an animal on and collecting data for the ICMS sensory discrimination paradigm.

We trained both animal groups to complete a series of progressively structured behavioral tasks, refining their ability to perform in a multi-stage two-choice sensory discrimination paradigm. Shown in Figure 3A, the basis of this paradigm consists of presenting a rat with one of two ICMS patterns where it could freely choose to nose poke in either the paired (correct) or unpaired (incorrect) nose-poke hole to receive a sugar pellet reward. If the unpaired hole was chosen, then the animal was delivered a mild air-puff punishment. Here, our study was divided into three phases: a “Training Phase” for initial task recognition, an “Experimental Phase” for evaluating discrimination proficiency, and a “Retraining Phase” to facilitate adaptation to new ICMS patterns which would then repeat until all desired comparisons were fulfilled. Training timelines and typical phase durations are outlined in Figure 3B. During the Training Phase, animals followed a four-tier protocol—Habituation, Shaping, Shape2Discriminate, and Discrimination—designed to condition them to associate a specific ICMS pattern with one of two nose-poke modules in exchange for sugar pellet rewards. The Experimental Phase then evaluated an animal’s ability to accurately discriminate between pairs of ICMS patterns. When introducing new patterns, the Retraining Phase reinstated elements of the Training Phase in a truncated format before resuming Experimental Phase trials. With every Retraining Phase cycle, Table 1 shows how we progressively reduced the spatial distance between ICMS pattern pairs to determine the limits of location-based sensory discrimination. Behavioral sessions were typically conducted 4 days per week, with an additional day allocated for neural recording data collection.

**Table 1:** Retraining phase ICMS pattern comparison order and corresponding center-to-center spatial distances.

Experimental group	ICMS pattern comparison order and spatial distances				
	First	Second	Third	Fourth	Fifth
Single-Shank	#1 vs. #5 (1,200 μm)	#1 vs. #4 (800 μm)	#1 vs. #3 (400 μm)	#1 vs. #2 (200 μm)	#1 vs. #1 (0 μm)
Four-Shank	#1 vs. #4 (375 μm)	#1 vs. #3 (250 μm)	#1 vs. #2 (125 μm)	#1 vs. #1 (0 μm)	N/A

#### 2.4.1 Training phase

Before pattern training, subjects underwent a one-week habituation period during which we handled them for a minimum of 10 cumulative hours or until they were able to at least tolerate head restraining for two consecutive minutes without struggle. This process ensured that once implanted, the animals could be safely connected to the stimulator without damaging the adapter or MEA connector. During habituation, we provided reward pellets to promote reward-seeking actions while also acclimating them to a conditioned reward for behavior.

Following habituation, the animals advanced to a one-hour daily Shaping task, in which they were rewarded with pellets for nose poking. Table 2 provides a detailed breakdown of the Training Phase’s session task routines. For a Shaping session, the rats were free to explore the behavioral chamber while the nose-poke modules alternated between “open” and “closed” states. This alternating procedure was designed to help acclimate the animals to the stepper motor functions and minimize side preference biases. At the start of each session, the chamber’s RGB lights illuminated green, signaling the beginning of an active trial. Alternatively, if the animal nose-poked in an available hole, a reward pellet would be dispensed, and the chamber lights would briefly transition to white for a three-second inter-trial period where additional nose pokes were ignored. To prevent accidental interactions in later training phases, a 150 ms delay period was introduced at the start of each trial before any nose pokes would register. If necessary, we administered manual reward pellets to encourage task engagement. Proficiency in this task was defined as successfully earning 100 + reward pellets in two consecutive sessions. Once this criterion was met, the animal then qualified for MEA implantation surgery.

**Table 2:** Training phase session task routines.

Training phase routines					
Training task	Stage	Duration	Module access	ICMS pattern probability	LED trial indicators and durations
Shaping (x2)	1	10 min	Both	N/A	Active Trial (Inf.*, green) Inter-Trial (3 s, white)
	2	10 min	Left only	N/A	Active Trial (Inf.*, green) Inter-Trial (3 s, white)
	3	10 min	Right only	N/A	Active Trial (Inf.*, green) Inter-Trial (3 s, white)
Shape2Discriminate	1	5 min	Left only	100%	Active Trial (6 s, green) Inter-Trial (3 s, white)
	2	5 min	Right only	100%	Active Trial (6 s, green) Inter-Trial (3 s, white)
	3	10 min	Left only	100%	Active Trial (6 s, green) Inter-Trial (3 s, white) Timeout (4 s, red)
	4	10 min	Right only	100%	Active Trial (6 s, green) Inter-Trial (3 s, white) Timeout (4 s, red)
	5	30 min	Both	50/50%	Active Trial (6 s, green) Inter-Trial (3 s, white) Timeout (8 s, red)
Discriminate	1	60 min	Both	50/50%	Active Trial (6 s, green) Inter-Trial (3 s, white) Timeout (8 s, red)

*The active trial duration was not constrained during this time, giving the animal the entire session duration to poke if it chose to.

After a one-week recovery period, the animals began Shape2Discriminate training, where they were conditioned to nose-poke in response to distinct ICMS patterns at naïve threshold values. The single-shank group was trained to discriminate between patterns #1 vs. #5, while the four-shank group learned patterns #1 vs. #4. These opposing patterns were introduced first to maximize the probability of a discriminable sensation before gradually transitioning to closer pattern pairs in subsequent Retraining Phases (see Table 1). In this paradigm, pattern #1 served as a control stimulus, consistently assigned to the left nose-poke module throughout the study. Meanwhile, the right nose-poke module featured a rotating ICMS pattern, which was systematically replaced with progressively closer patterns over time. This approach required the animals to learn only one new pattern per Retraining Phase, streamlining the adaptation process. Similar to the Shaping task, green lights indicated active trial periods, while white lights signaled inter-trial periods. However, trial durations were now limited to 6 s, and nose-poke access rotated in three progressive stages shown in Table 2. To refine task performance, incorrect responses now resulted in a mild air puff to the nose followed by a delay period signaled by red lights (timeout sequence); additional nose pokes during a timeout would extend the duration of the delay period. Air puff intensity was adjusted for each animal, with some requiring higher pressures to be effective, while others did not require it at all. Overall, these stages were designed to first reinforce pattern associations, then introduce mild punishments to discourage incorrect responses, and finally test comprehension under free-choice conditions. Proficiency in this task was achieved once animals obtained 100 + session pellets and maintained at least 70% accuracy during stages 2 and 3 for three consecutive sessions. Here, accuracy was defined as the percentage of trials where the animal nose-poked in the correct module when presented with its corresponding ICMS pattern. Using the computer vision methods developed previously (Smith et al., 2024b), individual trials were excluded when an animal was found to be “distracted” during the entirety of a trial—characterized by the animal facing away from the chamber’s module wall and/or its head remaining in the opposing half of the chamber from the module wall.

Finally, the Discrimination task mirrored that of the Shape2Discriminate’s third stage, except now it was present throughout the entire hour-long session. Here, proficiency was maintained at a 70% + accuracy score for three consecutive sessions. After passing, the animals proceeded to their first Experimental Phase.

#### 2.4.2 Experimental phase

**Figure 4:**
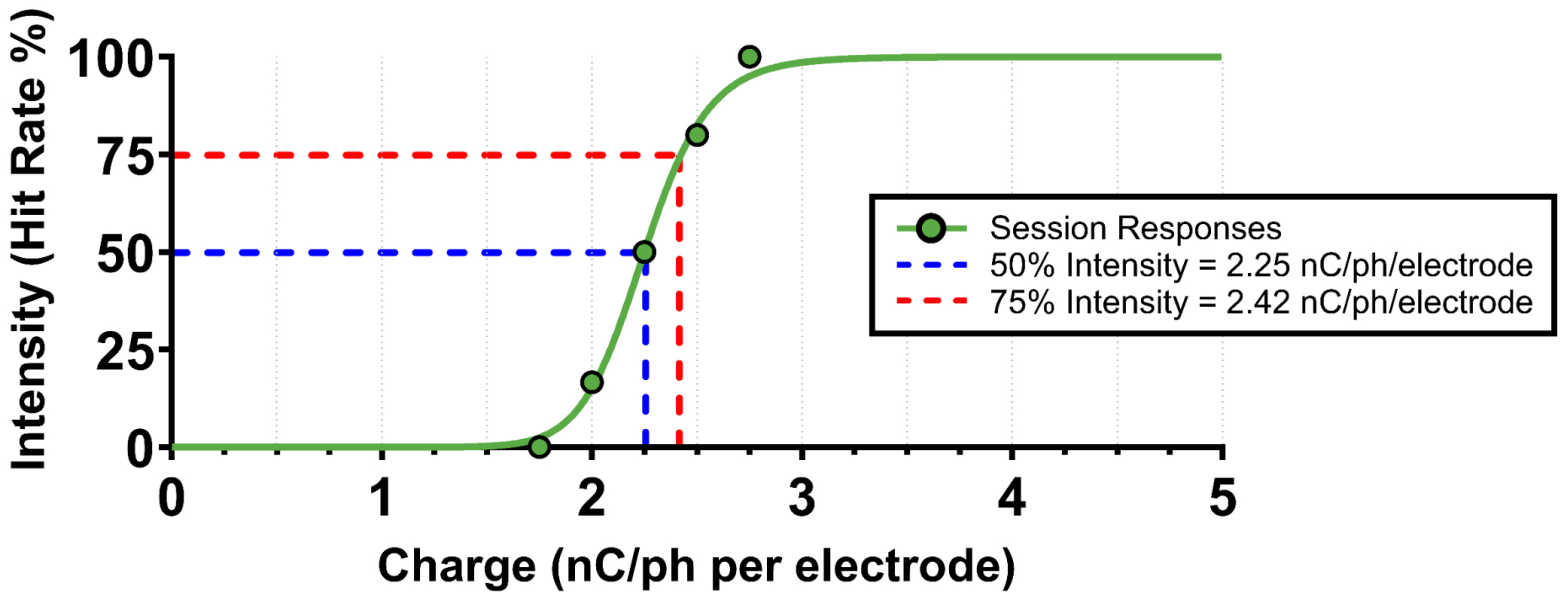
Example of ICMS pattern intensity normalization. Following every Go/No-Go session, ICMS pattern threshold magnitudes (at 50% hit rate) were calculated using a non-linear regression psychometric curve fit. The 75% intensity values (at 75% hit rate) were then extracted from the curve for normalization between ICMS patterns. An example from a singular Go/No-Go session is shown above.

Each Experimental Phase spanned 4 days and consisted of two steps. On the first day, each animal completed two Go/No-Go style tasks—one for each ICMS pattern—to establish pattern-specific perception threshold magnitudes. Perception thresholds have been observed to vary over time and differ based on cortical depth and location (Urdaneta et al., 2021; Urdaneta et al., 2022). To account for these variations, we normalized stimulus intensities across patterns by setting each to a stimulus magnitude that corresponded with a 75% hit rate value on a stimulus–response based psychometric curve (Figure 4). Specifically, when an animal was presented with that particular charge value, it had a 75% chance of perceiving the stimulus and nose poking. The 75% intensity level ensured consistent responses to the presented stimuli, unlike 50% (at threshold), where responses would be unreliable. Furthermore, this level was chosen over the 100% hit rate value to avoid saturating levels of current that may cause electrode damage.

Threshold estimations were determined from a 17-min adaptation of the Go/No-Go task described in Smith et al. (2023). Briefly, we dynamically adjusted stimulus amplitudes in the Go/No-Go task using a modified up/down staircase method, beginning at the naïve threshold intensity for each pattern. Throughout the session, charge magnitudes increased or decreased based on the animal’s responses, refining threshold estimates throughout the session. To prevent predictability, naïve threshold, dynamic stimulus, and catch (no stimulus) trials were each presented with equal probabilities (33.3%) starting after a 2-min period of 50/50% between naïve and catch trials. Moreover, since the animals were not initially trained in this Go/No-Go paradigm, air puffs were administered for false alarms and repeated pokes. If needed, Go/No-Go sessions could be repeated until the animal scored at least a 75% accuracy for the session. After each session, the dynamic stimulus trials were extracted and plotted on a stimulus–response based psychometric curve to calculate threshold values (Smith et al., 2023).

Since threshold magnitudes may change over time (Urdaneta et al., 2022) and were only estimated from a single session per pattern, the normalization process could have inherent variability between sessions. To account for this, slight adjustments were made to the presented charge values based on the animal’s responses. If the ICMS elicited an excessively strong reaction, the intensity was reduced, provided the animal continued to respond reliably. Conversely, if the stimulus was too weak—evidenced by a lack of nose poking for at least 10 trials per session—the charge was increased. In both instances, the session would be repeated with the adjusted values.

After estimating threshold magnitudes for each pattern, the second step of the Experimental Phase consisted of three standard Discrimination task sessions. In these sessions, ICMS charge values were set to the newly calculated 75% intensity levels, and session durations were reduced to 30 min per day.

#### 2.4.3 Retraining phase

Retraining Phases served as a way of efficiently comparing new ICMS patterns to the control without needing to repeat the entire training process. To achieve this, each Retraining Phase consisted of a truncated one-week Training Phase followed by a standard Experimental Phase (see Figure 3). The truncated Training Phase incorporated a naïve threshold evaluation of the new pattern, two sessions of the Shape2Discriminate task, and then two sessions of the Discriminate task. Afterward, the Go/No-Go threshold evaluation task of the Experimental Phase was repeated only for the new ICMS pattern, as the control pattern’s value was previously determined. Overall, this routine allowed us to evaluate a new pattern every 2 weeks while retaining animal performance despite the introduction of a new stimulus.

### 2.5 Computational modeling

Utilizing the biophysically realistic rhesus macaque somatosensory cortex model detailed in Kumaravelu et al. (2022), we simulated our five single-shank ICMS patterns to assess the spatial distinctiveness of neural activation throughout the cortex. Here, ICMS current point sources were introduced for each electrode site within a pattern at the center of the simulated cortical volume measuring 400 μm × 400 μm × 2000 μm deep, based on dimensions originally set by the authors of the model (Kumaravelu et al., 2022). Stimulation parameters matched a single ICMS pulse corresponding to the in-vivo experiments, with current amplitudes derived from each pattern’s respective average 75% perception threshold intensity magnitude. Neuronal activation via antidromic propagation of axons—set at a depolarization threshold of −20 mV—was cumulatively determined throughout an 8 ms duration from the start of stimulus presentation. This duration was chosen not only to align with the predefined model parameters but also to minimize the size of accumulated simulation data and improve computational efficiency by reducing the time required to run the model. All simulations were computed using The University of Texas at Dallas’s high-performance computing cluster, Ganymede, which provided 16,000 CPU cores and over 800 GPUs optimized for large-scale neural modeling. Following simulation, the exported axon activation data was analyzed in MATLAB to quantify the spatial overlap between stimulation patterns accumulated during various durations. Data accumulated at durations 0.2, 0.5, and 8 ms (the end of the simulation) following ICMS initiation were reported to capture the effects at stimulation onset, the end of the ICMS pulse, and over a prolonged period to visualize propagation dynamics. This analysis was critical for determining whether the cortical activation fields associated with each ICMS pattern were sufficiently distinct to support discrimination, or if overlap in neural recruitment may have contributed to non-discriminable responses.

### 2.6 Statistical analysis

Experimental Phase Go/No-Go and Discrimination task data for each ICMS pattern were compiled into GraphPad Prism for comparison. For the Go/No-Go perception threshold data, we used a Shapiro–Wilk test to assess normality for each ICMS pattern’s average 50, 75%, and “adjusted” charge magnitude values. We then used a D’Agostino and Pearson test to assess the normality of each Discrimination task’s average accuracy and reaction time metrics followed by examination of their corresponding QQ plots to evaluate the results. For normally distributed data, we used repeated measures one-way ANOVAs with post-hoc Tukey tests to control for Type I errors during individual pairwise comparisons between pattern pair threshold, discrimination accuracy, and reaction time metrics. Alternatively, we used a Friedman test paired with a Dunn’s test for multiple comparisons as a non-parametric alternative to repeated-measures ANOVA and Tukey tests when normality cannot be assumed; this approach has been used in a similar intracortical microstimulation study (Kim et al., 2015a,b). We reported all normally distributed metrics as mean ± SEM and non-normal metrics with median and interquartile ranges. Statistical significance for this study was determined at *p < 0.05.

## 3 Results

In this study, we established a repeatable methodology for evaluating the discriminability of two ICMS-evoked sensations and identified the potential spatial limits of this discrimination from both a depth and adjacent column-like approach. We then compared our *in-vivo* single-shank results with computational modeling to better understand the possible factors influencing these limits. From the eight animals introduced into this study, one rat from each group was excluded from the results due to desensitization to the control stimulus which exceeded the maximum charge limit set before study completion. Following exclusions, *n* = 3 remained for each group.

### 3.1 ICMS intensity magnitudes

**Figure 5:**
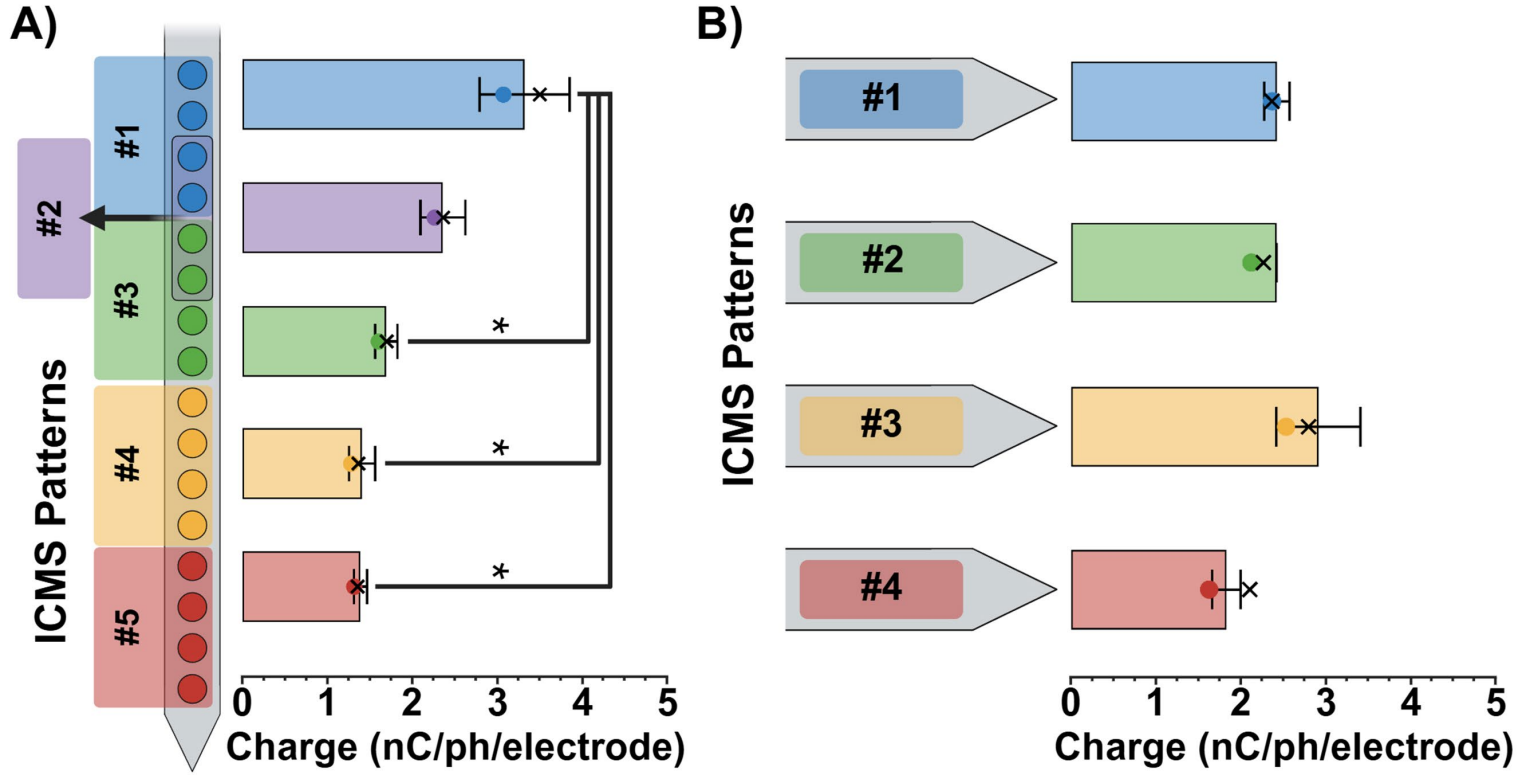
ICMS pattern intensity magnitudes. Average 75% intensity magnitudes (± SEM) for the single-shank **(A)** and four-shank **(B)** microelectrode array groups. Colored dots on each bar indicate the average 50% intensity magnitudes, while X’s represent the average adjusted magnitude values for each pattern. Results from repeated measures ANOVA with multiple comparisons are shown for each plot; statistical significance was set at *p < 0.05.

To ensure consistent stimulus intensities across ICMS patterns, perception threshold magnitudes were estimated using the Go/No-Go task and then normalized to a 75% intensity level. For the single-shank patterns, we found that the average 75% intensity magnitudes ± SEM ranged from 3.3 ± 0.5 nC/ph/electrode for the most superficial pattern (pattern #1; 450–750 μm) to 1.4 ± 0.1 nC/ph/electrode for the deepest (pattern #5; 1,650–1950 μm), decreasing in magnitude as the patterns got deeper (Figure 5A). A one-way repeated-measures ANOVA revealed a significant effect of pattern separation depth on 75% intensity magnitude values [*F*(4, 8) = 14.2, *p* = 0.0011]. Additionally, post-hoc Tukey comparisons found that the 75% intensity magnitude value for patterns #3, #4, and #5 were significantly lower than the superficial pattern #1 (*p* < 0.05). No other significant differences were observed between pattern pairs. In contrast, the four-shank patterns ranged from 2.9 ± 0.5 nC/ph/electrode to 1.8 ± 0.2 nC/ph/electrode, remaining relatively consistent for all four patterns (Figure 5B). A one-way repeated-measures ANOVA [*F*(3, 6) = 2.56, *p* = 0.1519] and *post hoc* Tukey comparisons (*p* > 0.05) revealed no significant effect of lateral pattern separation on 75% intensity magnitude values between ICMS patterns, displaying consistency at similar depths. See Supplementary Tables 1, 2 for each animal’s estimated 50 and 75% intensity charge magnitude values as well as their “adjusted” magnitudes used during every experimental session.

### 3.2 Discrimination task

**Figure 6:**
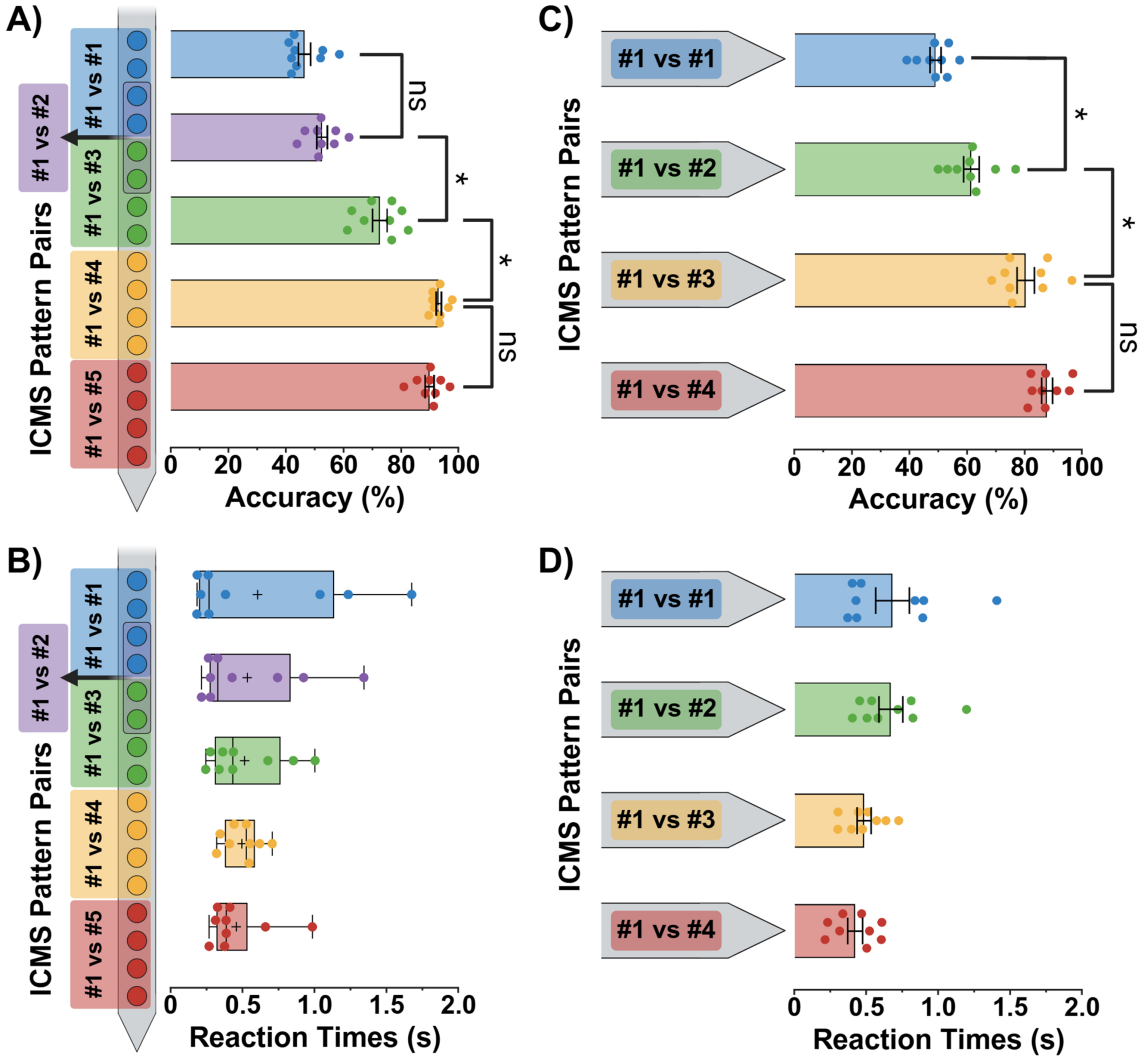
Discrimination task performance metrics. Average accuracy and reaction time performance metrics (± SEM) for the single-shank **(A,B)** and four-shank **(C,D)** microelectrode array groups. Since the single-shank group’s reaction time data **(B)** did not pass a normality test, it is presented as the median with interquartile ranges and max/min error bars; plus signs indicate the mean value for each pattern. Results from repeated measure statistical tests with multiple comparisons are shown for each plot. In plots **(A,C)**, all additional pattern pair comparisons were also significant, but only the most relevant are displayed for clarity. In contrast, no comparisons reached significance for plots **(B,D)**. Statistical significance was set at *p < 0.05.

We evaluated each animal’s ability to distinguish between two spatially separated ICMS patterns by measuring their average discrimination task accuracy and reaction times for all single-shank and four-shank pattern pair comparisons (Figure 6).

#### 3.2.1 Single-shank performance

In the single-shank group, results from a total of 3,423 trials (685 average trials per pattern pair) were compiled to calculate performance metrics with only 82 trials excluded due to lack of apparent engagement in the paradigm by the subject. Overall, a one-way repeated-measures ANOVA revealed a significant effect of pattern separation depth on discrimination accuracy [*F*(4, 32) = 134.1, *p* < 0.0001]. Additionally, a post-hoc Tukey comparison found that discrimination accuracy significantly decreased as the spatial separation between ICMS patterns was reduced below a center-to-center pattern depth distance of 800 μm (*p* < 0.05). This is shown in Figure 6A with ICMS pattern pair comparisons (#1 vs. #5 and #1 vs. #4) possessing high discrimination accuracy scores of 89.9 ± 1.6% (mean ± SEM) and 93.1 ± 0.9%, while closer pattern pairs (#1 vs. #3 and #1 vs. #2) held moderate to low accuracy scores of 72.6 ± 2.5% and 52.6 ± 1.8%, respectively. Furthermore, the ~53% accuracy shown for pattern pair #1 vs. #2 was not statistically significant from the control (pattern #1) vs. itself, providing evidence of random nose-poking and indistinguishable stimuli at spatial differences ≤ 200 μm.

Reaction times for the single-shank group (Figure 6B) were analyzed using a Friedman test due to non-normal data distribution (*p* < 0.05, D’Agostino-Pearson test). Additionally, Friedman and Dunn’s multiple comparison tests revealed that no pattern pair reaction times were statistically significant from one another (Friedman statistic = 0.8889, *p* = 0.9261; *p* > 0.05). In the end, we found that the longest pattern pair reaction time was pattern #1 vs. #4 with a median value 0.53 s (IQR: 0.38–0.59 s). In contrast, the shortest time was for pattern #1 vs. itself with a median of 0.27 s (IQR: 0.20–1.14 s).

#### 3.2.2 Four-shank performance

In the four-shank group, results from a total of 2,987 trials (747 average trials per pattern pair) were collected to calculate performance metrics with 46 trials excluded due to lack of apparent engagement in the paradigm by the subject. Overall, a one-way repeated-measures ANOVA revealed a significant effect of cortical column pattern separation on discrimination accuracy [*F*(3, 24) = 48.0, *p* < 0.0001]. Additionally, a post-hoc Tukey comparison found that discrimination accuracy significantly decreased as the spatial separation between ICMS patterns was reduced below a cortical column pattern distance of 250 μm (*p* < 0.05). This is shown in Figure 6C with ICMS pattern pair comparisons (#1 vs. #4 and #1 vs. #3) possessing high discrimination accuracy scores of 88.0 ± 1.9% (mean ± SEM) and 80.6 ± 3.0%, while the adjacent pattern pair (#1 vs. #2) held a low accuracy score of 61.6 ± 2.7%, respectively. Furthermore, the ~62% accuracy shown for pattern pair #1 vs. #2 was still statistically significant from the control (pattern #1) vs. itself, indicating that the indistinguishable limit was not yet reached and must be less than a cortical column spatial difference of 125 μm.

Reaction times for the four-shank group (Figure 6D) remained relatively stable across all pattern comparisons. Although a repeated-measures ANOVA test did find a significant difference in reaction times between pattern pairs [*F*(3, 24) = 3.8, *p* = 0.0242], the Tukey multiple comparison tests did not find any significant differences, indicating that they were fairly similar from one to another (*p* > 0.05). We found the longest average reaction time ± SEM to be 0.68 ± 0.12 s for control pattern (pattern #1) vs. itself, while the shortest was 0.42 ± 0.05 s for pattern #1 vs. #4.

### 3.3 Computational modeling

**Figure 7:**
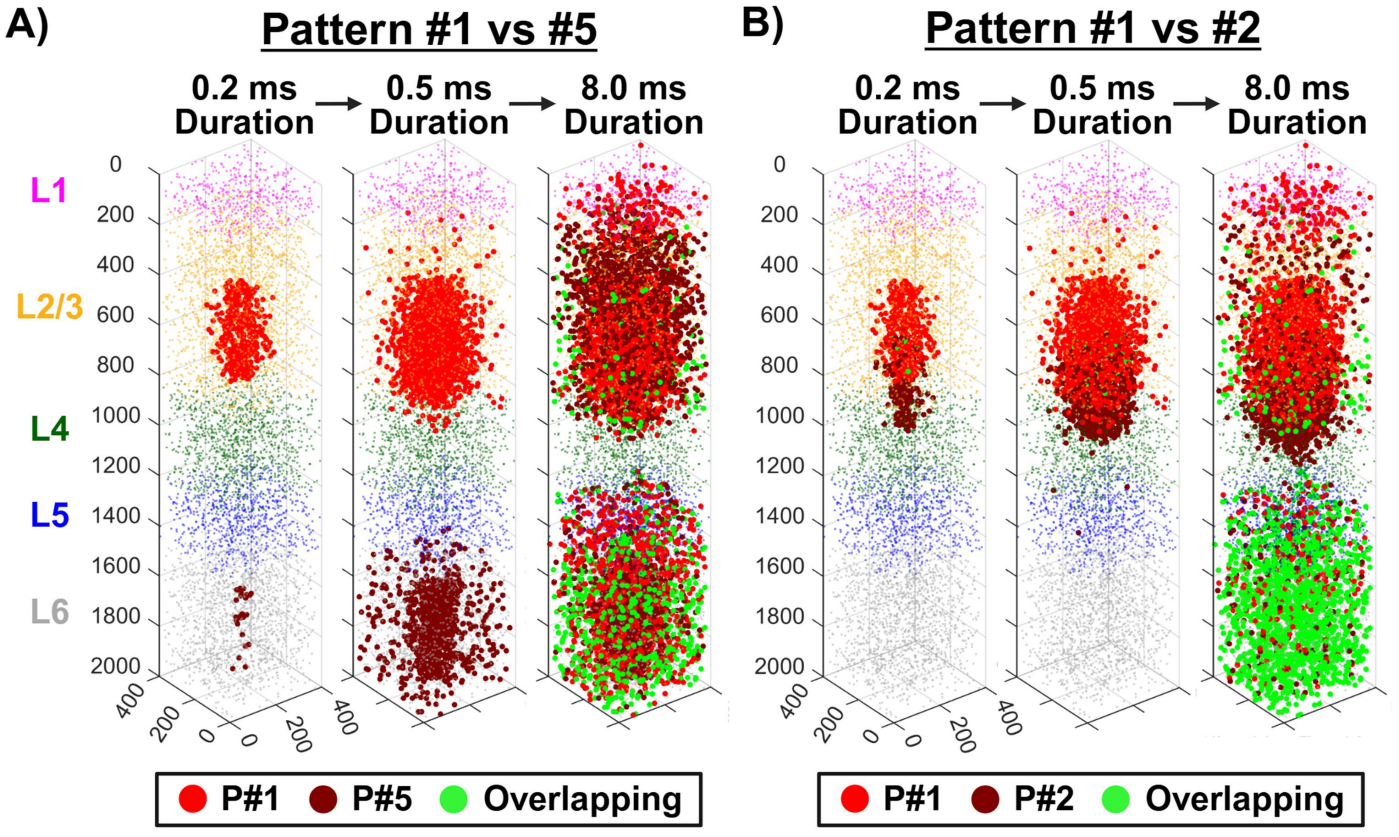
Modeled responses to ICMS across different stimulation patterns in single-shank devices. Simulated axonal activation sites (larger red/green dots) indicate where ICMS initiated an action potential for patterns #1 (P#1) vs. #5 (P#5) in panel **(A)** and patterns #1 vs. #2 (P#2) in panel **(B)**. These activations are shown at simulation durations of 0.2, 0.5, and 8 ms following pulse initiation. Green dots denote overlapping activation locations between the two patterns. Following axonal activation, action potentials propagated antidromically toward the soma.

To assess the spatial extent of neuronal activation induced by ICMS, we applied a biophysically realistic computational model of the somatosensory cortex to simulate neuronal recruitment across different stimulation patterns. In Figure 7, patterns #1, #2, and #5 were selected for visualization based on behavioral discrimination outcomes, with pattern pair #1 vs. #5 representing the most spatially distinct pair that exhibited high discriminability performance, and pattern #1 vs. #2 representing the closest pair that showed poor discrimination accuracy. Axonal activation location counts were quantified at simulation durations of 0.2, 0.5, and 8 ms following ICMS pulse initiation. In a broad qualitative examination of the plots, pattern #1 vs. #5 (Figure 7A) maintained distinct activation volumes during early time points, whereas pattern #1 vs. #2 (Figure 7B) exhibited a high degree of overlap, reflecting their spatial proximity. Quantitatively, pattern #1 (16.5 μA per electrode) displayed axonal activation increases from 418 locations at 0.2 ms to 2,529 at 0.5 ms and 5,415 at 8 ms. When comparing pattern #1 vs. #5 (7 μA per electrode), pattern #5 activated only 26 locations at 0.2 ms, increasing to 1,279 at 0.5 ms and 4,483 at 8 ms. Notably, no overlapping activations were observed at the shorter durations, but 1,016 locations (10%) overlapped with pattern #1 by the 8 ms time point, showing the effects of propagation across layers. In contrast, for pattern #1 vs. #2 (12 μA per electrode), pattern #2 activated 257 locations at 0.2 ms, 2,348 at 0.5 ms, and 5,115 at 8 ms. Overlapping locations between the two patterns were minimal at 0.2 ms with only 18 locations (3%) but grew to 314 (6%) at 0.5 ms and 2,194 (21%) at 8 ms, demonstrating greater overlap throughout the stimulation period than pattern #5. Overall, these results suggest that spatially distinct patterns remained more separable in activation volume, while closely spaced patterns exhibited substantial activation overlap, potentially limiting their discriminability.

## 4 Discussion

In this study, we investigated the spatial limits of ICMS-evoked sensory discrimination in the somatosensory cortex by comparing spatially separated depth-dependent and adjacent columnar stimulation patterns. Here, our results demonstrated a general trend in which discrimination accuracy declined as the spatial distance between stimulation patterns decreased. In addition, perception thresholds varied across depth, with deeper ICMS patterns requiring lower charge magnitudes to reach robust detection, consistent with findings from other rodent studies (Urdaneta et al., 2021; Kunigk et al., 2022; Urdaneta et al., 2022). For the single-shank group, we showed that our rats were able to discriminate between the most superficial and deepest patterns with high accuracy but struggled to differentiate between the closely spaced patterns, particularly for the pattern pair comparison of #1 vs. #2 which was separated by a center-to-center pattern distance of 200 μm. This decline in accuracy suggests that ICMS-evoked percepts at adjacent depths may activate overlapping neural populations, limiting perceptual differentiation along the depth axis. Computational modeling supported this interpretation, as simulations revealed that axonal activation volumes for pattern #1 vs. #2 showed substantial overlap, whereas pattern #1 vs. #5 remained spatially distinct. Future work may explore whether discrimination performance is a function of pattern separation or if the corresponding cortical layers, which were not addressed in this study, affect separability. In addition to the single-shank group, the four-shank animals demonstrated similar performance results, but with better spatial resolutions. We found that they were still capable of discriminating between ICMS patterns 250 μm apart with high accuracies above 80% and, to some degree, discriminate between patterns that were only 125 μm apart with ~60% accuracy. Although we acknowledge that the similar electrode-site depths of pattern #5 in the single-shank group may have served as a better control for comparative procedures against the four-shank group, our results suggest that spatial resolution for discriminable ICMS percepts may be more constrained along cortical depths than across adjacent cortical columns. However, future clinical studies are needed to investigate these findings.

Altogether, the single-shank discrimination limits were surprising. As cortical neurons have been shown to be organized into columns (Armstrong-James, 1975; Mountcastle, 1997; Geyer et al., 1999; Narayanan et al., 2017; Kumaravelu et al., 2022), we expected that stimulating different depths within a presumed single column of tissue would activate functionally related populations. And although the nature of the percepts that subjects registered in our studies is unclear, unlike other studies which have explored ICMS in human subjects (Kim et al., 2015b; Greenspon et al., 2024; Verbaarschot et al., 2024), the high accuracy scores in the present study provide evidence that animal subjects can reliably register differences between pattern pairs longitudinally within a column. Conversely, the four-shank results were more expected, as prior clinical studies using Utah arrays with 400 μm pitch spacings have routinely demonstrated robust somatotopic differentiations across the body (Verbaarschot et al., 2024; Valle et al., 2025). However, it was notable that our animals were still able to discriminate patterns separated by only 250 μm with high accuracy, a distance nearly half that of the typical Utah array pitch of 400 μm (Flesher et al., 2016; Armenta Salas et al., 2018; Hughes et al., 2021a,b). However, it should be noted that we did utilize simultaneous multi-channel stimulation within a single column, which may alter perceptual performance when compared to single-channel stimulation (Kunigk et al., 2022).

A key advantage of this behavioral paradigm when paired with computational modeling is in its flexibility for evaluating several alternative stimulation parameters. Differences in frequency, pulse width, and amplitude could each be substituted for the spatial differences outlined in this study, allowing for a broader exploration of the neural mechanisms underlying sensory encoding. This flexibility also opens the possibility of exploring alternative stimulation modes. For example, future studies could evaluate bipolar stimulation configurations, which may reduce current spread and enhance discriminability compared to the monopolar stimulation used in this study (Schelles et al., 2025). However, investigational limitations do exist for our approach. First, the number of stimulus conditions that can be evaluated in this paradigm is ultimately constrained by the stability and longevity of the implanted arrays. As we noted in the results, two of our animals became desensitized to our control stimuli and therefore excluded from the study. Although we did not confirm the root cause of this desensitization, biological, mechanical, and material-based failures often occur at the electrode-tissue interface (Prasad et al., 2014; Campbell and Wu, 2018; Woeppel et al., 2021; Bjånes et al., 2024). In response to these dropouts, pilot histological data was collected for the four-shank group. Supplementary Figure 1 shows immunohistochemistry results for two 100 μm transverse cortical brain slices from a cortical depth of ~1.6 mm which were stained with NeuN for identification of neurons and GFAP for glial cells such as astrocytes. While shank holes were clearly visible at a depth of 1.6 mm, it was difficult to visualize depths relevant to the electrode sites themselves, which were located slightly deeper (1.65–1.95 mm). This limitation is consistent with previous studies (Abbott et al., 2024; Haghighi et al., 2025), demonstrating incomplete visualization of deeper tracks in transverse sections of cortical tissue. In addition, the established NeurostimML program (Li et al., 2024), designed to predict if a set of ICMS parameters was likely to be damaging or non-damaging when applied to neural tissue, indicated that our ICMS was “likely not damaging.”

Another limitation lies in the sequential order of pattern testing, which could have contributed to performance declines due to insufficient training time on new patterns. Although Figure 6 shows that subsequent pattern comparisons following the initial discrimination tests were not statistically different in accuracy or reaction time (i.e., patterns pairs #1 vs. #5 against #1 vs. #4 in the single-shank group and pattern pairs #1 vs. #4 against #1 vs. #3 in the four-shank group), it remains possible that animals experienced increasing task difficulty due to carryover effects during the retraining stages. However, similar behavioral paradigms which sequentially train rats to nose poke on variations of auditory stimuli demonstrate that learning consecutive discrimination targets does not impede nor improve task acquisition or performance accuracy (Carroll et al., 2024). Furthermore, changes in discriminable accuracy may be partially impacted by the differential variability of ICMS sensitivity. We demonstrate in Figure 5 that perception thresholds decrease with depth for the single-shank device from the most superficial to the deepest ICMS patterns by approximately half. Although we attempt to normalize intensity magnitudes to control for this confound, further testing with more animals is needed to determine if ICMS magnitude is sufficient as the only normalized ICMS parameter compared to other properties such as frequency (Kim et al., 2015a). Finally, the physical spacing between electrode sites constrained the resolution of our spatial discrimination measurements. While we identified an indistinguishable pattern pair for the single-shank group with patterns #1 vs. #2, the four-shank study did not reach a definitive spatial cutoff where performance dropped to chance. Future studies should explore denser electrode arrays with smaller inter-site and inter-shank pitches to further refine discriminable limits.

Overall, these findings establish a methodology for systematically evaluating the limits of ICMS-evoked sensory discrimination, alongside implementation of a biophysically realistic computational model of the somatosensory cortex to better interpret its results. Ultimately, our work will provide valuable insight for the design of future microelectrode arrays aimed at enabling selective sensory perception via ICMS.

## 5 Conclusion

In summary, this study establishes a novel methodology for evaluating the spatial limits of ICMS-evoked sensory discrimination in rodent somatosensory cortex by presentation of a two-choice sensory discrimination task controlled via expandable/retractable nose-poke module holes. Our findings demonstrate that discrimination accuracy declines as the spatial separation between stimulation patterns decreases, with more pronounced limitations across cortical depths compared to adjacent cortical columns. For the laminar probe, animal subjects could robustly differentiate between stimuli separated by 800 μm along a cortical column whereas, for the multi-shank probes, animal subjects could robustly differentiate between stimuli delivered from shanks separated by 250 μm. These results highlight the role of spatially distinct neural activation volumes in sensory encoding and have important implications for the design of future neuroprosthetic devices. Additionally, our pre-clinical paradigm provides a robust and flexible framework for future investigations into ICMS-based sensory discrimination performance, including comparisons of alternative stimulation parameters. Further studies incorporating finer electrode spacing and more randomized testing procedures will be essential to refine our understanding of the neural mechanisms underlying ICMS-evoked perceptual discrimination.

## Data Availability

The raw data supporting the conclusions of this article will be made available by the authors, without undue reservation.
