# Supplementary material for: Investigating the spatial limits of somatotopic and depth-dependent sensory discrimination stimuli in rats via intracortical microstimulation
Source: Front Neurosci. 2025 May 14;19:1602996. doi: 10.3389/fnins.2025.1602996 (PMC12116559; doi:10.3389/fnins.2025.1602996)
Supplement: Supplementary file 1 [file Data_Sheet_1.docx]

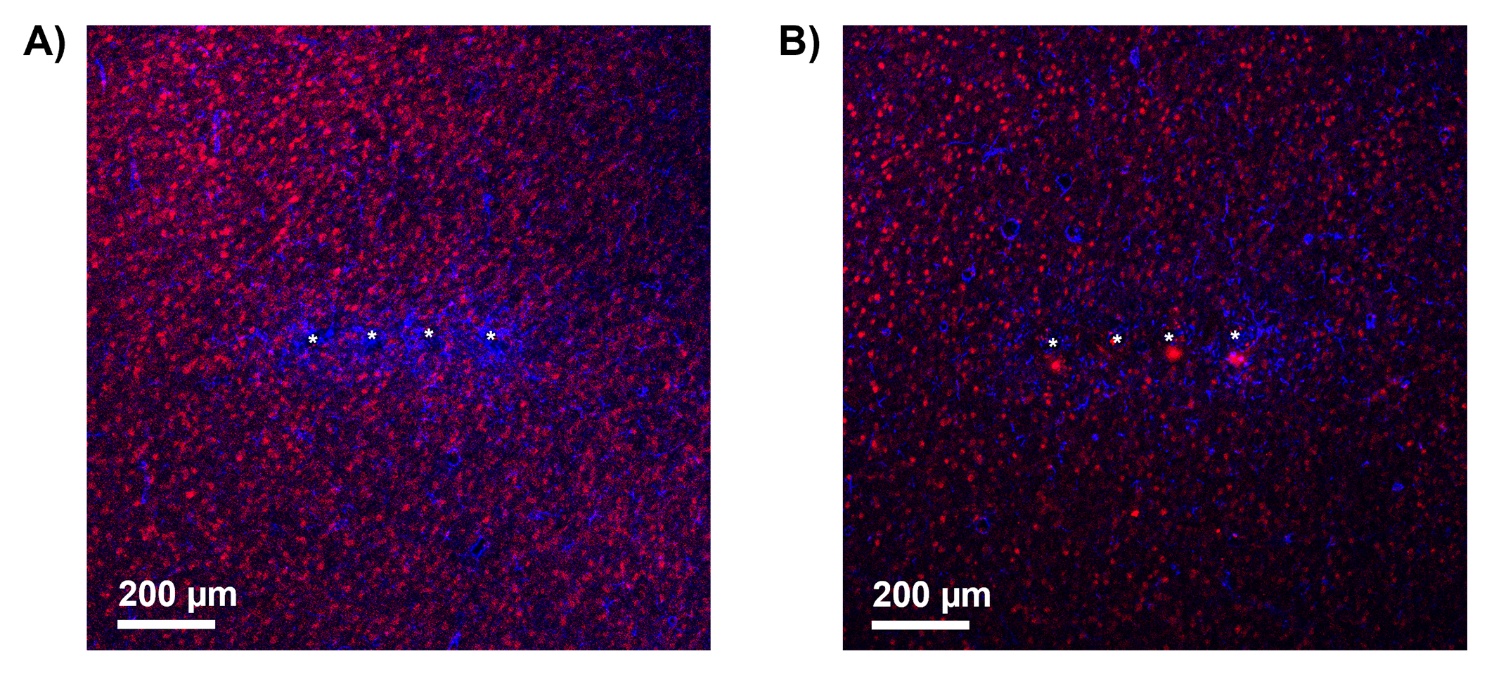


Supplementary Figure 1. Pilot immunohistochemical tissue analysis of the four-shank implanted ICMS animal group showing 10x transversely sectioned z-stack images of a 100 µm thick somatosensory cortex slice located ~1.6 mm deep from the cortical surface. Panel (A) contains an image from the single excluded four-shank animal, whereas panel (B) displays a representative image from a four-shank animal who had finished the study. In both images, cells were stained for NeuN (red) and GFAP (blue). Residual shank holes were indicated with an asterisk.

Supplemental Table 1. Charge magnitudes (nC/ph/electrode) recorded for the 50% intensity, 75% intensity, and ‘adjusted’ values across all Experimental Phase sessions for the single-shank animal group. Since pattern #1 served as the control stimulus for all comparisons, multiple values are listed per session.

| Single-Shank Group | | | | | | | | | | | | | | | | | | |
| --- | --- | --- | --- | --- | --- | --- | --- | --- | --- | --- | --- | --- | --- | --- | --- | --- | --- | --- |
|  | ICMS Pattern | 50% | 75% | Experimental Session(s) #1 | | | | | Experimental Session(s) #2 | | | | | Experimental Session(s) #3 | | | | |
| Animal #1 | #1 | 2.81 | 3.13 | 3.50 | 3.50 | 3.50 | 4.00 | 4.00 | 3.75 | 3.50 | 3.50 | 4.00 | 4.00 | 3.50 | 3.50 | 3.50 | 4.00 | 4.00 |
|  | #2 | 2.60 | 2.73 | 2.75 | | | | | 2.75 | | | | | 2.75 | | | | |
|  | #3 | 1.87 | 1.88 | 2.00 | | | | | 2.00 | | | | | 2.00 | | | | |
|  | #4 | 1.52 | 1.66 | 1.70 | | | | | 1.70 | | | | | 1.70 | | | | |
|  | #5 | 1.19 | 1.40 | 1.50 | | | | | 1.30 | | | | | 1.30 | | | | |
| Animal #2 | #1 | 3.91 | 4.33 | 4.00 | 4.00 | 4.00 | 4.00 | 4.00 | 4.00 | 4.00 | 4.00 | 4.00 | 4.00 | 4.00 | 4.00 | 4.00 | 4.00 | 4.00 |
|  | #2 | 2.50 | 2.51 | 2.50 | | | | | 2.50 | | | | | 2.50 | | | | |
|  | #3 | 1.76 | 1.77 | 1.80 | | | | | 1.80 | | | | | 1.80 | | | | |
|  | #4 | 1.36 | 1.44 | 1.40 | | | | | 1.40 | | | | | 1.40 | | | | |
|  | #5 | 1.51 | 1.52 | 1.50 | | | | | 1.50 | | | | | 1.50 | | | | |
| Animal #3 | #1 | 2.51 | 2.52 | 2.50 | 2.50 | 3.00 | 3.00 | 3.00 | 2.50 | 2.50 | 3.00 | 3.00 | 3.00 | 2.50 | 2.50 | 3.00 | 3.00 | 3.00 |
|  | #2 | 1.70 | 1.85 | 1.85 | | | | | 1.85 | | | | | 1.85 | | | | |
|  | #3 | 1.19 | 1.44 | 1.30 | | | | | 1.30 | | | | | 1.30 | | | | |
|  | #4 | 0.96 | 1.13 | 1.00 | | | | | 1.00 | | | | | 1.00 | | | | |
|  | #5 | 1.25 | 1.25 | 1.20 | | | | | 1.20 | | | | | 1.20 | | | | |

Supplemental Table 2. Charge magnitudes (nC/ph/electrode) recorded for the 50% intensity, 75% intensity, and ‘adjusted’ values across all Experimental Phase sessions for the four-shank animal group. Since pattern #1 served as the control stimulus for all comparisons, multiple values are listed per session.

| Four-Shank Group | | | | | | | | | | | | | | | |
| --- | --- | --- | --- | --- | --- | --- | --- | --- | --- | --- | --- | --- | --- | --- | --- |
|  | ICMS Pattern | 50% | 75% | Experimental Session(s) #1 | | | | Experimental Session(s) #2 | | | | Experimental Session(s) #3 | | | |
| Animal #1 | #1 | 1.72 | 2.03 | 2.00 | 2.25 | 2.25 | 2.25 | 2.00 | 2.25 | 2.25 | 2.25 | 2.00 | 2.25 | 2.25 | 2.25 |
|  | #2 | 3.53 | 3.82 | 3.80 | | | | 3.80 | | | | 3.80 | | | |
|  | #3 | 1.97 | 2.42 | 2.00 | | | | 2.00 | | | | 2.00 | | | |
|  | #4 | 2.12 | 2.13 | 2.10 | | | | 2.10 | | | | 2.10 | | | |
| Animal #2 | #1 | 1.49 | 1.50 | 1.50 | 2.00 | 2.50 | 2.50 | 1.50 | 2.00 | 2.50 | 2.50 | 1.50 | 2.00 | 2.50 | 2.50 |
|  | #2 | 1.78 | 2.11 | 2.10 | | | | 2.10 | | | | 2.10 | | | |
|  | #3 | 2.25 | 2.42 | 2.40 | | | | 2.40 | | | | 2.40 | | | |
|  | #4 | 2.62 | 2.63 | 2.50 | | | | 2.50 | | | | 2.50 | | | |
| Animal #3 | #1 | 1.67 | 1.96 | 2.00 | 2.00 | 2.00 | 2.00 | 2.00 | 2.00 | 2.00 | 2.00 | 2.00 | 2.00 | 2.00 | 2.00 |
|  | #2 | 2.29 | 2.82 | 2.50 | | | | 2.50 | | | | 2.50 | | | |
|  | #3 | 2.15 | 2.42 | 2.40 | | | | 2.40 | | | | 2.40 | | | |
|  | #4 | 2.37 | 2.52 | 2.50 | | | | 2.50 | | | | 2.50 | | | |
